# Scorecard Rates Emission Reductions of Hybrid Vehicles

**DOI:** 10.1289/ehp.119-a384a

**Published:** 2011-09-01

**Authors:** David C. Holzman

**Affiliations:** David C. Holzman writes on science, medicine, energy, economics, and cars from Lexington and Wellfleet, MA. His work has appeared in *Smithsonian*, *The Atlantic Monthly*, and the *Journal of the National Cancer Institute*.

For all its cachet, you might think that hybrid drivetrain technology is inherently green. But only 13 of 34 hybrid vehicles assessed achieve better than a 25% reduction in greenhouse gas (GHG) emissions, and just 3 exceed a 40% reduction, according to an evaluation by the Union of Concerned Scientists (UCS).^1^ Moreover, reductions in GHG emissions do not necessarily correlate with reductions in other toxic emissions.

Like any engine output–improving technology, hybrid technology can boost both fuel efficiency and power—but the more you boost one, the less you can boost the other. That dichotomy spurred the UCS to develop its “hybrid scorecard,” which rates each hybrid according to how well it lives up to its promise of reducing air pollution.^2^ All the vehicles were from model year 2011 except for one, the 2012 Infiniti M Hybrid.

First the UCS scored each hybrid on how much it reduced its GHG emissions relative to its conventional counterpart, on a scale of zero (least reduction) to 10 (greatest reduction). These scores reflect the percentage in fuel efficiency gain. For example, the Toyota Prius gets 50 mpg^3^ compared with 28 mpg for the comparable Toyota Matrix. This represents a 44.0% reduction in GHG emissions, earning the Prius a GHG score of 9.4. At the bottom of the scale, the 21-mpg hybrid VW Touareg reduces GHG emissions only 10% over the 19-mpg conventional Toureg, for a score of 0.0. With a 46% improvement, the luxury Lincoln MKZ Hybrid had the greatest reduction over its conventional counterpart.

The UCS also scored hybrids for absolute emissions (rather than relative to the conventional model) of air pollutants including particulate matter, carbon monoxide, hydrocarbons, and nitrogen oxides. These scores, on a scale of zero (dirtiest) to 10 (cleanest), are based on California certifications for tailpipe emissions. As the scorecard showed, a vehicle that emits less heat-trapping gases may not necessarily emit less of other air pollutants. For example, the Mercedes Benz S400 Hybrid scored 9 on air pollution reduction, alongside the Prius and the Lincoln MKZ, but only 1.3 on GHG emissions.

**Figure:**
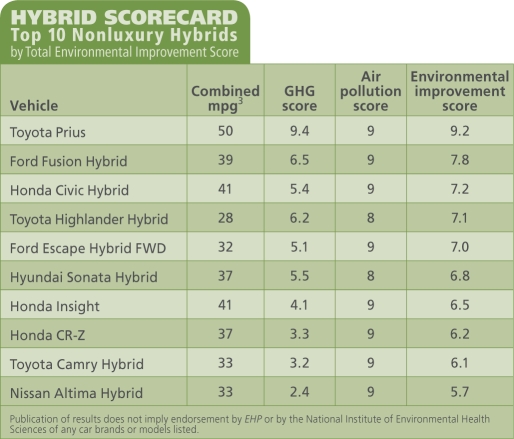
HYBRID SCORECARD: Top 10 Nonluxury Hybrids by Total Environmental Improvement Score

“Hybrid technology doesn’t add additional challenges [to reducing exhaust pollutants] that can’t be addressed through design of the vehicle’s emission controls,” says Don Anair, senior vehicles analyst at the UCS. “Numerous manufacturers of hybrids are meeting the lowest emissions levels. Hybrid manufacturers who aren’t delivering the lowest smog-forming emissions have chosen not to do so.”

Each vehicle’s air pollution and GHG scores were averaged into a total “environmental improvement score,” again with the MKZ and the Prius leading the pack, and the Touareg scraping bottom. The UCS also scored “hybrid value” (the cost of reducing GHG emissions in dollars per percent reduction) and “forced features” (options you must buy with the hybrid whether you want them or not).

**Figure:**
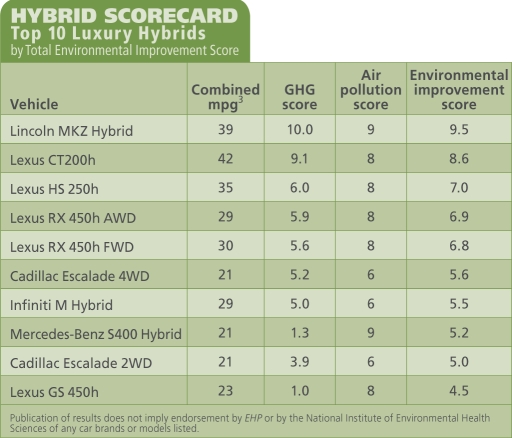
HYBRID SCORECARD: Top 10 Luxury Hybrids by Total Environmental Improvement Score

Luke Tonachel, vehicles analyst with the Natural Resources Defense Council, compliments the scorecard for illustrating that hybrid technology is not automatically green. He says, “We should improve the efficiency of all vehicles, and [hybrid technology] is just one technology that can get us there if applied with that goal in mind.”

Nonetheless, Jamie Kitman, the New York bureau chief for *Automobile Magazine*, questions the wisdom of emphasizing percentage improvement in gas mileage rather than absolute miles per gallon. At 21 mpg, the hybrid Cadillac Escalade 4WD represents a 29% improvement over the 15-mpg conventional model, saving nearly 2 gallons per 100 miles. But the hybrid Escalade is still a gas guzzler, and Kitman says he wishes people would see through the marketing that encourages them to buy SUVs and “crossovers” rather than ordinary cars, which are more efficient than either.

Says Anair, “The scorecard shows that automakers can pair hybrid technology with advanced emission controls to help tackle climate change while reducing the health impacts from breathing polluted air.” However, he adds, alluding to the stark variation in how much hybrid technology boosted fuel efficiency, “Not all automakers are delivering on the full promise of this technology.”
